# Supplementary material for: Takotsubo cardiomyopathy in patients suffering from acute non-traumatic subarachnoid hemorrhage—A single center follow-up study
Source: PLoS One. 2022 May 26;17(5):e0268525. doi: 10.1371/journal.pone.0268525 (PMC9135260; doi:10.1371/journal.pone.0268525)
Supplement: S1 Table — (DOCX) [file pone.0268525.s001.docx]

**Supplemental Table 1.** Occurrence of TTC by comorbidities of the patients

| **Comorbidity** | **No TTC**  **N=97** | **TTC**  **N=39** | **Yates χ^2^** |
| --- | --- | --- | --- |
| Hypertension (Yes/No) | 54/43 | 19/20 | 0.297 n.s. |
| Arrhytmias or conduction disturbances (Yes/No) | 3/94 | 1/38 | 0.000 n.s. |
| Hypercholesterinemia/trigliceridaemia (Yes/No) | 11/86 | 4/35 | 0.000 n.s. |
| Diabetes mellitus (Yes/No) | 4/93 | 1/38 | 0.000 n.s. |
| Hypothyreosis (Yes/No) | 3/94 | 0/39 | 0.000 n.s. |
| Hyperthyreosis (Yes/No) | 0/97 | 1/38 | 0.224 n.s. |
| Smoking (Yes/No) | 47/50 | 19/20 | 0.000 n.s. |
| Obesity (Yes/No) | 26/71 | 7/32 | 0.754 n.s. |
