# Supplementary material for: Takotsubo cardiomyopathy in patients suffering from acute non-traumatic subarachnoid hemorrhage—A single center follow-up study
Source: PLoS One. 2022 May 26;17(5):e0268525. doi: 10.1371/journal.pone.0268525 (PMC9135260; doi:10.1371/journal.pone.0268525)
Supplement: S2 Table — (DOCX) [file pone.0268525.s002.docx]

**Supplemental Table 2.** Severity of TTC by comorbidities of the patients

| **Comorbidity** | **No TTC**  **N=97** | **Moderate TTC**  **N=28** | **Severe TTC**  **N=11** | **χ^2^** |
| --- | --- | --- | --- | --- |
| Hypertension (Yes/No) | 54/43 | 14/14 | 5/6 | 0.606 n.s. |
| Arrhytmia or conduction disturbance (Yes/No) | 3/94 | 1/27 | 0/11 | 0.380 n.s. |
| Hypercholesterin/  Hypertrigliceridaemia (Yes/No) | 11/86 | 2/26 | 2/9 | 1.014 n.s. |
| Diabetes mellitus (Yes/No) | 4/93 | 0/28 | 1/10 | 2.034 n.s. |
| Hypothyreosis (Yes/No) | 3/94 | 0/28 | 0/11 | 1.233 n.s. |
| Hyperthyreosis (Yes/No) | 0/97 | 1/27 | 0/11 | 3.886 n.s. |
| Smoking (Yes/No) | 47/50 | 13/15 | 6/5 | 0.209 n.s. |
| Obesity (Yes/No) | 26/71 | 6/22 | 1/10 | 1.841 n.s. |
