# Supplementary material for: Takotsubo cardiomyopathy in patients suffering from acute non-traumatic subarachnoid hemorrhage—A single center follow-up study
Source: PLoS One. 2022 May 26;17(5):e0268525. doi: 10.1371/journal.pone.0268525 (PMC9135260; doi:10.1371/journal.pone.0268525)
Supplement: S3 Table — (DOCX) [file pone.0268525.s003.docx]

**Supplemental Table 3**. Results of general linear models testing the differences in normetenephrin concentration between study groups and the effect of arterenol and dobutrex on normetanephrin concentration on days 1 and 30.

| **Day** | **Effect** | **Coefficient ± S.E.** | **F (df_1_, df_2_)** | **p** |
| --- | --- | --- | --- | --- |
| Day 1 | Study group, control vs. moderate TTC | -216.0 ± 256.32 | 85.475 (2, 118) | p < 0.0001 |
|  | Study group, control vs. severe TTC | -95.5 ± 289.59 |  |  |
|  | Arterenol | 243.7 ± 16.56 | 256.888 (1,118) | p < 0.0001 |
|  | Dobutamin | 3.4 ± 1.44 | 5.545 (1,118) | p < 0.0001 |
| Day 30 | Study group, control vs. moderate TTC | -747.6 ± 352.80 | 2.576 (1,68) | p = 0.084 |
|  | Study group, control vs. severe TTC | -444.1 ± 374.6 |  |  |
|  | Arterenol | 111.8 ± 113.8 | 0.965 | 0.329 |
