# Supplementary material for: Takotsubo cardiomyopathy in patients suffering from acute non-traumatic subarachnoid hemorrhage—A single center follow-up study
Source: PLoS One. 2022 May 26;17(5):e0268525. doi: 10.1371/journal.pone.0268525 (PMC9135260; doi:10.1371/journal.pone.0268525)
Supplement: Attachment — Submitted filename: Response, revision.docx [file pone.0268525.s004.docx]

**Tomohiko Ai, M.D., Ph.D.
Academic Editor**

**PLOS ONE**

**Dear Editor,**

Thank you very much for giving us the opportunity to revise our manuscript entitled „Takotsubo Cardiomyopathy in Patients Suffering From Acute Non-traumatic Subarachnoid Hemorrhage - a Single Center Follow-up Study”.

In the following w erespond point-by point to the comments of the reviewer. The changes in the manuscript are marked with red color.

***Comments from the editorial office***

- **Funding:** Hungarian Brain research program [grant number KTIA_13_NAP-A-II/5], the founder is the Hungarian Academy of Sciences. The funders had no role in study design, data collection and analysis, decision to publish, or preparation of the manuscript.
- **Data availability:** In the declarations section we stated: „all available material (including raw data and statistical analysis will be made available upon request from the corresponding author.”
- **phrase “data not shown”:** we delated this part from the text as suggested.
- **Supporting Information files:** we included them at the end of the manuscript, as suggested.

***Reviewer and editor’s comment***

- **Novelty of the study as compared to previous reports.** Thank you for this comment. For sake of clarity we added a section to the discussion section that is intended to point out the novelties of the present study. This part of the discussion sections reads now as follows: „The present report is among the first prospective studies assessing the incidence, predisposing factors and long-term clinical outcome of patients with TTC after aneurysmal subarachnoid hemorrhage (aSAH) in a parallel, complex fashion. Our findings contribute to better understanding the natural history of TTC after aSAH and suggests a link between pathophysiological factors, clinical presentation of TTC and outcome parameters. As all aSAH patients were consecutively included, the reported incidence in our cohort reflects the real incidence of TTC according to recent recommendations. An important novelty is the systematic, prospective, long-term (180 days) follow up of the echocardiographic parameters in parallel with the cardiac biomarkers and time-collected urine catecholamine concentrations. Furthermore, based on the predefined ejection fraction threshold of 40%, we were able to define mild and severe forms of TTC in the acute phase of subarachnoid hemorrhage that showed a good relationship with long term outcome of the patients. We were the first demonstrating that cardiac wall motion abnormalities in severe TTC cases may persist for as long as 6 months after SAH onset.”

Again, thank you very much for the thorough review process and for your suggestions. We hope that in its present form the manuscript will meet the requirements of the journal.

Yours sincerely:

Béla Fülesdi

Professor and chairman

Department of Anesthesiology and IC
